# 5-(4-Fluoro­phen­yl)-3-(4-methyl­phen­yl)-4,5-dihydro-1*H*-pyrazole-1-carbothio­amide

**DOI:** 10.1107/S1600536813004194

**Published:** 2013-02-16

**Authors:** Bakr F. Abdel-Wahab, Hanan A. Mohamed, Rizk E. Khidre, Seik Weng Ng, Edward R. T. Tiekink

**Affiliations:** a Applied Organic Chemistry Department, National Research Centre, Dokki, 12622 Giza, Egypt; b Chemical Industries Division, National Research Centre, Dokki, 12622, Giza, Egypt; c Department of Chemistry, University of Malaya, 50603 Kuala Lumpur, Malaysia; d Chemistry Department, Faculty of Science, King Abdulaziz University, PO Box 80203 Jeddah, Saudi Arabia

## Abstract

The central pyrazole ring in the title compound, C_17_H_16_FN_3_S, adopts an envelope conformation with the methine C atom bearing the 4-fluoro­phenyl substituent as the flap atom. Whereas the tolyl ring is slightly twisted out of the least-squares plane through the pyrazole ring [dihedral angle = 13.51 (11)°], the fluoro­benzene ring is almost perpendicular [dihedral angle = 80.21 (11)°]. The thio­amide group is almost coplanar with the N—N bond of the ring [N—N—C—N torsion angle = 1.2 (3)°] and the amine group forms an intra­molecular hydrogen bond with a ring N atom. In the crystal, supra­molecular double layers in the *bc* plane are formed *via* N—H⋯S, N—H⋯F and C—H⋯F inter­actions.

## Related literature

For the biological activity of pyrazolin-1-yl­thia­zoles, see: Abdel-Wahab *et al.* (2009 ▶, 2012 ▶); Chimenti *et al.* (2010 ▶). For related structures, see: Nonthason *et al.* (2011 ▶); Chantra­promma *et al.* (2012 ▶).
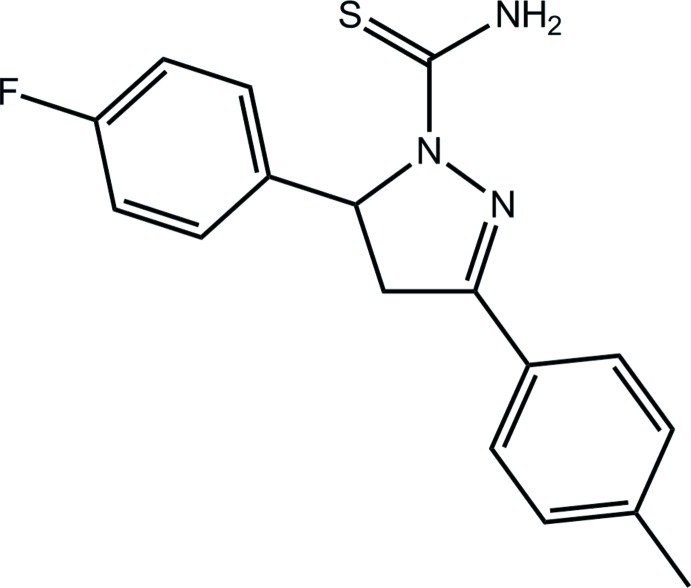


## Experimental

### 

#### Crystal data

C_17_H_16_FN_3_S
*M* *_r_* = 313.39
Monoclinic, 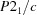

*a* = 14.4154 (10) Å
*b* = 11.3197 (9) Å
*c* = 9.5575 (8) Å
β = 103.991 (8)°
*V* = 1513.3 (2) Å^3^
*Z* = 4
Mo *K*α radiation
μ = 0.22 mm^−1^
*T* = 295 K
0.30 × 0.20 × 0.10 mm

#### Data collection

Agilent SuperNova Dual diffractometer with an Atlas detector
Absorption correction: multi-scan (*CrysAlis PRO*; Agilent, 2011 ▶) *T*_min_ = 0.772, *T*_max_ = 1.000
9303 measured reflections
3500 independent reflections
2370 reflections with *I* > 2σ(*I*)
*R*_int_ = 0.038

#### Refinement

*R*[*F* ^2^ > 2σ(*F* ^2^)] = 0.047
*wR*(*F* ^2^) = 0.122
*S* = 1.04
3500 reflections
200 parameters
H-atom parameters constrained
Δρ_max_ = 0.18 e Å^−3^
Δρ_min_ = −0.26 e Å^−3^

### 

Data collection: *CrysAlis PRO* (Agilent, 2011 ▶); cell refinement: *CrysAlis PRO*; data reduction: *CrysAlis PRO*; program(s) used to solve structure: *SHELXS97* (Sheldrick, 2008 ▶); program(s) used to refine structure: *SHELXL97* (Sheldrick, 2008 ▶); molecular graphics: *ORTEP-3 for Windows* (Farrugia, 2012 ▶) and *DIAMOND* (Brandenburg, 2006 ▶); software used to prepare material for publication: *publCIF* (Westrip, 2010 ▶).

## Supplementary Material

Crystal structure: contains datablock(s) global, I. DOI: 10.1107/S1600536813004194/hg5291sup1.cif

Structure factors: contains datablock(s) I. DOI: 10.1107/S1600536813004194/hg5291Isup2.hkl

Supplementary material file. DOI: 10.1107/S1600536813004194/hg5291Isup3.cml

Additional supplementary materials:  crystallographic information; 3D view; checkCIF report

## Figures and Tables

**Table 1:** Hydrogen-bond geometry (Å, °)

*D*—H⋯*A*	*D*—H	H⋯*A*	*D*⋯*A*	*D*—H⋯*A*
N3—H31⋯N2	0.88	2.23	2.611 (3)	106
N3—H31⋯F1^i^	0.88	2.41	3.255 (2)	162
N3—H32⋯S1^ii^	0.88	2.83	3.538 (2)	138
C16—H16*B*⋯F1^iii^	0.96	2.55	3.478 (3)	163

Symmetry codes: (i) 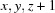; (ii) 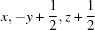; (iii) 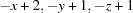.

**Fig. 1.**
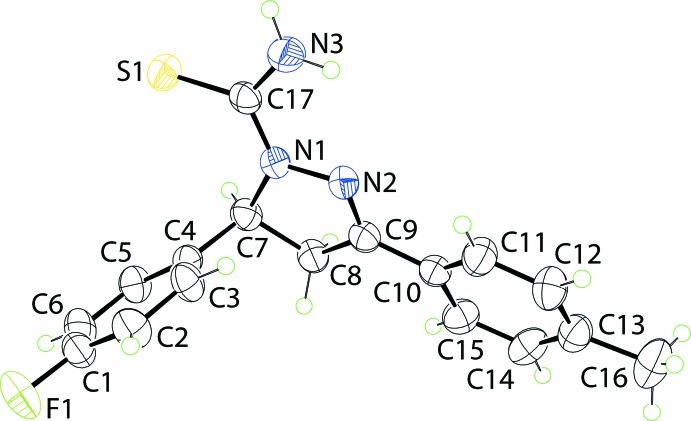
The molecular structure of (I) showing the atom-labelling scheme and displacement ellipsoids at the 50% probability level.

**Fig. 2.**
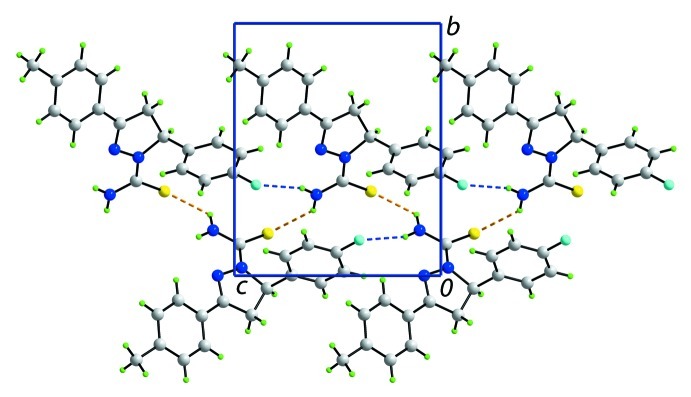
A view of the supramolecular chain along the c axis in (I) mediated by N—H···S and N—H···F hydrogen bonds, shown as orange and blue dashed lines, respectively.

**Fig. 3.**
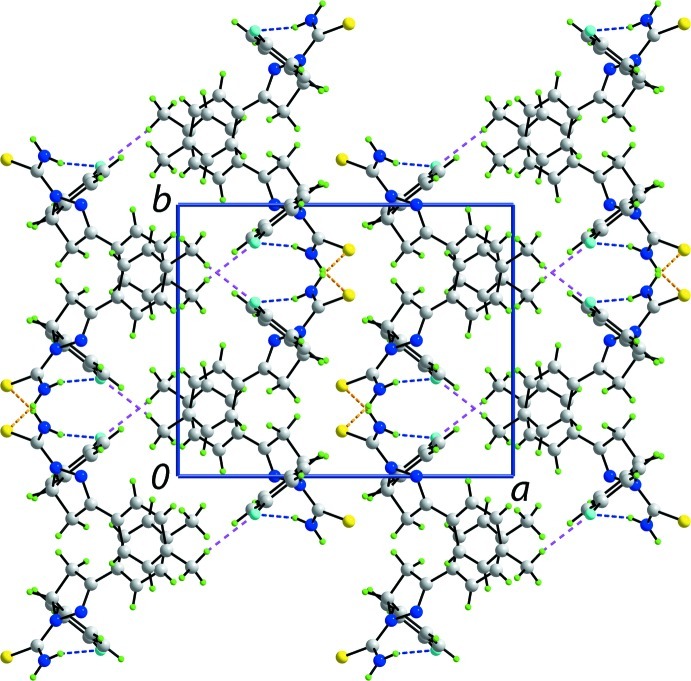
A view of the crystal packing in projection down the c axis. The N—H···S, N—H···F and C—H···F interactions are shown as orange, blue and pink dashed lines, respectively.

## supplementary crystallographic information

### Comment

The title compound (I) was investigated crystallographically in connection with the established biological activities exhibited by pyrazolin-1-ylthiazoles (Abdel-Wahab *et al.*, 2012; Abdel-Wahab *et al.*, 2009; Chimenti *et al.*, 2010).

The central pyrazolyl ring in (I), Fig. 1, adopts an envelope conformation with the methine-C7 atom being the flap atom. The tolyl ring is slightly twisted out of the least-squares plane through the pyrazolyl ring, forming a dihedral angle of 13.51 (11)°. By contrast, the fluorobenzene ring is almost perpendicular to the five-membered ring [dihedral angle = 80.21 (11)°]. Finally, the thioamide residue is co-planar with the N2—N1—C17—N3 torsion angle being 1.2 (3)°; the amine group is orientated towards the ring-N2 atom, forming a hydrogen bond, Table 1. Similar conformations have been observed in related structures bearing two six-membered rings (Nonthason *et al.*, 2011; Chantrapromma *et al.*, 2012).

The F atom proves to be pivotal in providing stability to the crystal structure of (I). Thus, in addition to forming an intramolecular N—H···N2 hydrogen bond, the amine-H31 atom forms a hydrogen bond to the F1 atom, Table 1. The second amine-H32 atom hydrogen bonds to a thione so that a supramolecular chain along the *c* axis ensues, Fig. 2. Chains stack along the *b* axis to form a row and centrosymmetrically related rows inter-digitate along the *a* axis allowing for the formation methylene-C16—H···F1 interactions and, therefore, double layers, Fig. 2.

### Experimental

A mixture of 5-(4-fluorophenyl)-3-*p*-tolyl-4,5-dihydro-1*H*-pyrazole-1-carbothioamide (0.31 g, 0.001 *M*) and 2-bromo-1-(4-chlorophenyl)ethanone (0.23 g, 0.001 *M*) in anhydrous ethanol (30 ml) was heated under reflux for about 4 h. The resultant solid was filtered and dried. Re-crystallization was by slow evaporation of DMF solution of (I) which yielded colourless crystals in 61% yield. *M*.pt: 513–514 K.

### Refinement

Nitrogen- and carbon-bound H-atoms were placed in calculated positions (N—H = 0.88 Å, and C—H 0.93 to 0.98 Å) and were included in the refinement in the riding model approximation, with *U*_iso_(H) = 1.2–1.5*U*_equiv_(N,*C*).

### Crystal data

**Table:**

C_17_H_16_FN_3_S	*F*(000) = 656
*M**_r_* = 313.39	*D*_x_ = 1.376 Mg m^−^^3^
Monoclinic, *P*2_1_/*c*	Mo *K*α radiation, λ = 0.71073 Å
Hall symbol: -P 2ybc	Cell parameters from 2000 reflections
*a* = 14.4154 (10) Å	θ = 2.9–27.5°
*b* = 11.3197 (9) Å	µ = 0.22 mm^−^^1^
*c* = 9.5575 (8) Å	*T* = 295 K
β = 103.991 (8)°	Prism, colourless
*V* = 1513.3 (2) Å^3^	0.30 × 0.20 × 0.10 mm
*Z* = 4	

### Data collection

**Table:**

Agilent SuperNova Dual diffractometer with an Atlas detector	3500 independent reflections
Radiation source: SuperNova (Mo) X-ray Source	2370 reflections with *I* > 2σ(*I*)
Mirror monochromator	*R*_int_ = 0.038
Detector resolution: 10.4041 pixels mm^-1^	θ_max_ = 27.6°, θ_min_ = 2.9°
ω scan	*h* = −18→17
Absorption correction: multi-scan (*CrysAlis PRO*; Agilent, 2011)	*k* = −10→14
*T*_min_ = 0.772, *T*_max_ = 1.000	*l* = −12→12
9303 measured reflections	

### Refinement

**Table:**

Refinement on *F*^2^	Primary atom site location: structure-invariant direct methods
Least-squares matrix: full	Secondary atom site location: difference Fourier map
*R*[*F*^2^ > 2σ(*F*^2^)] = 0.047	Hydrogen site location: inferred from neighbouring sites
*wR*(*F*^2^) = 0.122	H-atom parameters constrained
*S* = 1.04	*w* = 1/[σ^2^(*F*_o_^2^) + (0.0448*P*)^2^ + 0.2973*P*] where *P* = (*F*_o_^2^ + 2*F*_c_^2^)/3
3500 reflections	(Δ/σ)_max_ < 0.001
200 parameters	Δρ_max_ = 0.18 e Å^−^^3^
0 restraints	Δρ_min_ = −0.26 e Å^−^^3^

### Special details

**Table:**

Geometry. All e.s.d.'s (except the e.s.d. in the dihedral angle between two l.s. planes) are estimated using the full covariance matrix. The cell e.s.d.'s are taken into account individually in the estimation of e.s.d.'s in distances, angles and torsion angles; correlations between e.s.d.'s in cell parameters are only used when they are defined by crystal symmetry. An approximate (isotropic) treatment of cell e.s.d.'s is used for estimating e.s.d.'s involving l.s. planes.
Refinement. Refinement of *F*^2^ against ALL reflections. The weighted *R*-factor *wR* and goodness of fit *S* are based on *F*^2^, conventional *R*-factors *R* are based on *F*, with *F* set to zero for negative *F*^2^. The threshold expression of *F*^2^ > σ(*F*^2^) is used only for calculating *R*-factors(gt) *etc*. and is not relevant to the choice of reflections for refinement. *R*-factors based on *F*^2^ are statistically about twice as large as those based on *F*, and *R*- factors based on ALL data will be even larger.

### Fractional atomic coordinates and isotropic or equivalent isotropic displacement parameters (Å^2^)

**Table:**

	*x*	*y*	*z*	*U*_iso_*/*U*_eq_	
S1	0.48991 (4)	0.33372 (5)	0.33938 (6)	0.04739 (19)	
F1	0.76993 (11)	0.35963 (12)	−0.10409 (14)	0.0659 (4)	
N1	0.63664 (12)	0.46879 (14)	0.46526 (17)	0.0384 (4)	
N2	0.71143 (11)	0.50005 (15)	0.58117 (17)	0.0391 (4)	
N3	0.60198 (14)	0.32075 (15)	0.60342 (19)	0.0491 (5)	
H31	0.6497	0.3462	0.6727	0.059*	
H32	0.5684	0.2591	0.6181	0.059*	
C1	0.73573 (16)	0.40770 (19)	0.0050 (2)	0.0445 (5)	
C2	0.77815 (16)	0.3771 (2)	0.1434 (2)	0.0483 (6)	
H2	0.8306	0.3266	0.1637	0.058*	
C3	0.74165 (14)	0.42262 (19)	0.2528 (2)	0.0435 (5)	
H3	0.7697	0.4024	0.3479	0.052*	
C4	0.66378 (13)	0.49796 (17)	0.2226 (2)	0.0341 (4)	
C5	0.62418 (15)	0.52828 (18)	0.0812 (2)	0.0416 (5)	
H5	0.5728	0.5804	0.0602	0.050*	
C6	0.65938 (16)	0.48273 (19)	−0.0307 (2)	0.0469 (5)	
H6	0.6320	0.5025	−0.1262	0.056*	
C7	0.62560 (14)	0.55094 (17)	0.3424 (2)	0.0376 (5)	
H7	0.5584	0.5736	0.3066	0.045*	
C8	0.68480 (15)	0.65640 (18)	0.4168 (2)	0.0406 (5)	
H8A	0.7267	0.6865	0.3594	0.049*	
H8B	0.6439	0.7198	0.4347	0.049*	
C9	0.74100 (14)	0.60325 (18)	0.5553 (2)	0.0378 (5)	
C10	0.82031 (14)	0.65987 (18)	0.6583 (2)	0.0381 (5)	
C11	0.87678 (15)	0.5981 (2)	0.7726 (2)	0.0486 (6)	
H11	0.8658	0.5179	0.7826	0.058*	
C12	0.94914 (17)	0.6537 (2)	0.8718 (3)	0.0526 (6)	
H12	0.9867	0.6101	0.9470	0.063*	
C13	0.96669 (15)	0.7734 (2)	0.8614 (2)	0.0470 (5)	
C14	0.91224 (16)	0.8339 (2)	0.7453 (3)	0.0519 (6)	
H14	0.9243	0.9136	0.7342	0.062*	
C15	0.83985 (15)	0.7787 (2)	0.6447 (2)	0.0470 (5)	
H15	0.8041	0.8216	0.5673	0.056*	
C16	1.04181 (18)	0.8345 (2)	0.9752 (3)	0.0673 (8)	
H16A	1.0751	0.8910	0.9303	0.101*	
H16B	1.0864	0.7772	1.0264	0.101*	
H16C	1.0119	0.8744	1.0414	0.101*	
C17	0.58067 (14)	0.37547 (17)	0.4763 (2)	0.0365 (5)	

### Atomic displacement parameters (Å^2^)

**Table:**

	*U*^11^	*U*^22^	*U*^33^	*U*^12^	*U*^13^	*U*^23^
S1	0.0422 (3)	0.0508 (3)	0.0468 (3)	−0.0061 (3)	0.0063 (2)	−0.0054 (3)
F1	0.0882 (10)	0.0730 (9)	0.0427 (8)	0.0155 (8)	0.0279 (7)	−0.0051 (7)
N1	0.0401 (9)	0.0438 (10)	0.0305 (8)	−0.0056 (8)	0.0071 (7)	−0.0003 (7)
N2	0.0387 (9)	0.0462 (10)	0.0315 (9)	−0.0038 (8)	0.0066 (7)	−0.0025 (8)
N3	0.0545 (11)	0.0474 (10)	0.0437 (10)	−0.0086 (9)	0.0088 (9)	0.0077 (8)
C1	0.0549 (13)	0.0448 (12)	0.0370 (11)	−0.0018 (11)	0.0170 (10)	−0.0035 (10)
C2	0.0448 (12)	0.0556 (13)	0.0442 (12)	0.0135 (11)	0.0099 (10)	0.0022 (11)
C3	0.0438 (11)	0.0547 (13)	0.0296 (10)	0.0095 (11)	0.0043 (9)	0.0018 (10)
C4	0.0342 (10)	0.0357 (10)	0.0315 (10)	−0.0021 (9)	0.0062 (8)	0.0008 (8)
C5	0.0433 (11)	0.0431 (11)	0.0346 (11)	0.0075 (10)	0.0020 (9)	0.0040 (9)
C6	0.0594 (14)	0.0487 (12)	0.0283 (10)	0.0028 (11)	0.0023 (10)	0.0040 (9)
C7	0.0360 (10)	0.0428 (11)	0.0325 (10)	0.0030 (9)	0.0056 (8)	0.0044 (9)
C8	0.0455 (11)	0.0403 (11)	0.0360 (11)	0.0006 (10)	0.0100 (9)	−0.0010 (9)
C9	0.0370 (10)	0.0422 (11)	0.0359 (10)	0.0003 (10)	0.0124 (9)	−0.0027 (9)
C10	0.0362 (10)	0.0427 (11)	0.0372 (11)	−0.0007 (9)	0.0124 (9)	−0.0038 (9)
C11	0.0509 (13)	0.0447 (12)	0.0469 (13)	−0.0063 (11)	0.0052 (11)	−0.0013 (10)
C12	0.0521 (13)	0.0542 (14)	0.0449 (13)	0.0011 (12)	−0.0010 (11)	−0.0012 (11)
C13	0.0385 (11)	0.0510 (13)	0.0509 (13)	0.0004 (10)	0.0098 (10)	−0.0134 (11)
C14	0.0434 (12)	0.0413 (12)	0.0695 (16)	−0.0045 (11)	0.0106 (12)	−0.0052 (11)
C15	0.0423 (12)	0.0465 (12)	0.0513 (13)	0.0009 (10)	0.0093 (10)	0.0026 (11)
C16	0.0527 (14)	0.0653 (16)	0.0745 (18)	−0.0040 (13)	−0.0025 (13)	−0.0231 (14)
C17	0.0384 (10)	0.0378 (11)	0.0365 (11)	0.0033 (9)	0.0151 (9)	−0.0034 (9)

### Geometric parameters (Å, º)

**Table:**

S1—C17	1.679 (2)	C7—C8	1.538 (3)
F1—C1	1.369 (2)	C7—H7	0.9800
N1—C17	1.349 (2)	C8—C9	1.501 (3)
N1—N2	1.392 (2)	C8—H8A	0.9700
N1—C7	1.476 (2)	C8—H8B	0.9700
N2—C9	1.288 (3)	C9—C10	1.464 (3)
N3—C17	1.332 (2)	C10—C11	1.383 (3)
N3—H31	0.8800	C10—C15	1.386 (3)
N3—H32	0.8800	C11—C12	1.381 (3)
C1—C2	1.361 (3)	C11—H11	0.9300
C1—C6	1.367 (3)	C12—C13	1.386 (3)
C2—C3	1.379 (3)	C12—H12	0.9300
C2—H2	0.9300	C13—C14	1.377 (3)
C3—C4	1.383 (3)	C13—C16	1.505 (3)
C3—H3	0.9300	C14—C15	1.385 (3)
C4—C5	1.377 (3)	C14—H14	0.9300
C4—C7	1.509 (3)	C15—H15	0.9300
C5—C6	1.389 (3)	C16—H16A	0.9600
C5—H5	0.9300	C16—H16B	0.9600
C6—H6	0.9300	C16—H16C	0.9600
			
C17—N1—N2	119.99 (16)	C9—C8—H8B	111.3
C17—N1—C7	127.16 (16)	C7—C8—H8B	111.3
N2—N1—C7	112.76 (15)	H8A—C8—H8B	109.2
C9—N2—N1	107.83 (16)	N2—C9—C10	120.57 (18)
C17—N3—H31	120.0	N2—C9—C8	113.58 (17)
C17—N3—H32	120.0	C10—C9—C8	125.81 (18)
H31—N3—H32	120.0	C11—C10—C15	118.05 (19)
C2—C1—F1	118.6 (2)	C11—C10—C9	121.63 (19)
C2—C1—C6	123.1 (2)	C15—C10—C9	120.31 (19)
F1—C1—C6	118.25 (19)	C12—C11—C10	121.0 (2)
C1—C2—C3	118.5 (2)	C12—C11—H11	119.5
C1—C2—H2	120.7	C10—C11—H11	119.5
C3—C2—H2	120.7	C11—C12—C13	121.1 (2)
C2—C3—C4	120.74 (19)	C11—C12—H12	119.4
C2—C3—H3	119.6	C13—C12—H12	119.4
C4—C3—H3	119.6	C14—C13—C12	117.8 (2)
C5—C4—C3	118.76 (18)	C14—C13—C16	121.6 (2)
C5—C4—C7	120.31 (17)	C12—C13—C16	120.6 (2)
C3—C4—C7	120.86 (17)	C13—C14—C15	121.5 (2)
C4—C5—C6	121.39 (19)	C13—C14—H14	119.3
C4—C5—H5	119.3	C15—C14—H14	119.3
C6—C5—H5	119.3	C14—C15—C10	120.6 (2)
C1—C6—C5	117.44 (19)	C14—C15—H15	119.7
C1—C6—H6	121.3	C10—C15—H15	119.7
C5—C6—H6	121.3	C13—C16—H16A	109.5
N1—C7—C4	111.36 (16)	C13—C16—H16B	109.5
N1—C7—C8	100.38 (15)	H16A—C16—H16B	109.5
C4—C7—C8	113.31 (16)	C13—C16—H16C	109.5
N1—C7—H7	110.5	H16A—C16—H16C	109.5
C4—C7—H7	110.5	H16B—C16—H16C	109.5
C8—C7—H7	110.5	N3—C17—N1	115.16 (18)
C9—C8—C7	102.56 (16)	N3—C17—S1	122.98 (16)
C9—C8—H8A	111.3	N1—C17—S1	121.85 (15)
C7—C8—H8A	111.3		
			
C17—N1—N2—C9	−167.33 (17)	N1—N2—C9—C10	−179.64 (16)
C7—N1—N2—C9	9.5 (2)	N1—N2—C9—C8	2.3 (2)
F1—C1—C2—C3	−177.94 (19)	C7—C8—C9—N2	−12.1 (2)
C6—C1—C2—C3	0.9 (3)	C7—C8—C9—C10	169.92 (18)
C1—C2—C3—C4	−0.2 (3)	N2—C9—C10—C11	11.6 (3)
C2—C3—C4—C5	−1.0 (3)	C8—C9—C10—C11	−170.55 (19)
C2—C3—C4—C7	−178.1 (2)	N2—C9—C10—C15	−167.37 (19)
C3—C4—C5—C6	1.5 (3)	C8—C9—C10—C15	10.5 (3)
C7—C4—C5—C6	178.58 (19)	C15—C10—C11—C12	1.4 (3)
C2—C1—C6—C5	−0.4 (3)	C9—C10—C11—C12	−177.6 (2)
F1—C1—C6—C5	178.42 (19)	C10—C11—C12—C13	0.9 (4)
C4—C5—C6—C1	−0.8 (3)	C11—C12—C13—C14	−2.7 (4)
C17—N1—C7—C4	−79.3 (2)	C11—C12—C13—C16	176.3 (2)
N2—N1—C7—C4	104.12 (18)	C12—C13—C14—C15	2.3 (3)
C17—N1—C7—C8	160.42 (18)	C16—C13—C14—C15	−176.7 (2)
N2—N1—C7—C8	−16.1 (2)	C13—C14—C15—C10	−0.1 (3)
C5—C4—C7—N1	149.56 (18)	C11—C10—C15—C14	−1.8 (3)
C3—C4—C7—N1	−33.4 (2)	C9—C10—C15—C14	177.28 (19)
C5—C4—C7—C8	−98.2 (2)	N2—N1—C17—N3	1.2 (3)
C3—C4—C7—C8	78.8 (2)	C7—N1—C17—N3	−175.12 (18)
N1—C7—C8—C9	15.56 (19)	N2—N1—C17—S1	−179.70 (14)
C4—C7—C8—C9	−103.26 (18)	C7—N1—C17—S1	4.0 (3)

### Hydrogen-bond geometry (Å, º)

**Table:**

*D*—H···*A*	*D*—H	H···*A*	*D*···*A*	*D*—H···*A*
N3—H31···N2	0.88	2.23	2.611 (3)	106
N3—H31···F1^i^	0.88	2.41	3.255 (2)	162
N3—H32···S1^ii^	0.88	2.83	3.538 (2)	138
C16—H16*B*···F1^iii^	0.96	2.55	3.478 (3)	163

Symmetry codes: (i) *x*, *y*, *z*+1; (ii) *x*, −*y*+1/2, *z*+1/2; (iii) −*x*+2, −*y*+1, −*z*+1.
